# Supplementary material for: TNF alpha unmasks enteric malate aspartate shuttle dysfunction bridging Parkinson disease and intestinal inflammation
Source: Nat Commun. 2026 Apr 1;17:3217. doi: 10.1038/s41467-026-71317-y (PMC13057073; doi:10.1038/s41467-026-71317-y)

Supplemental information

Supplementary figures and figure legends

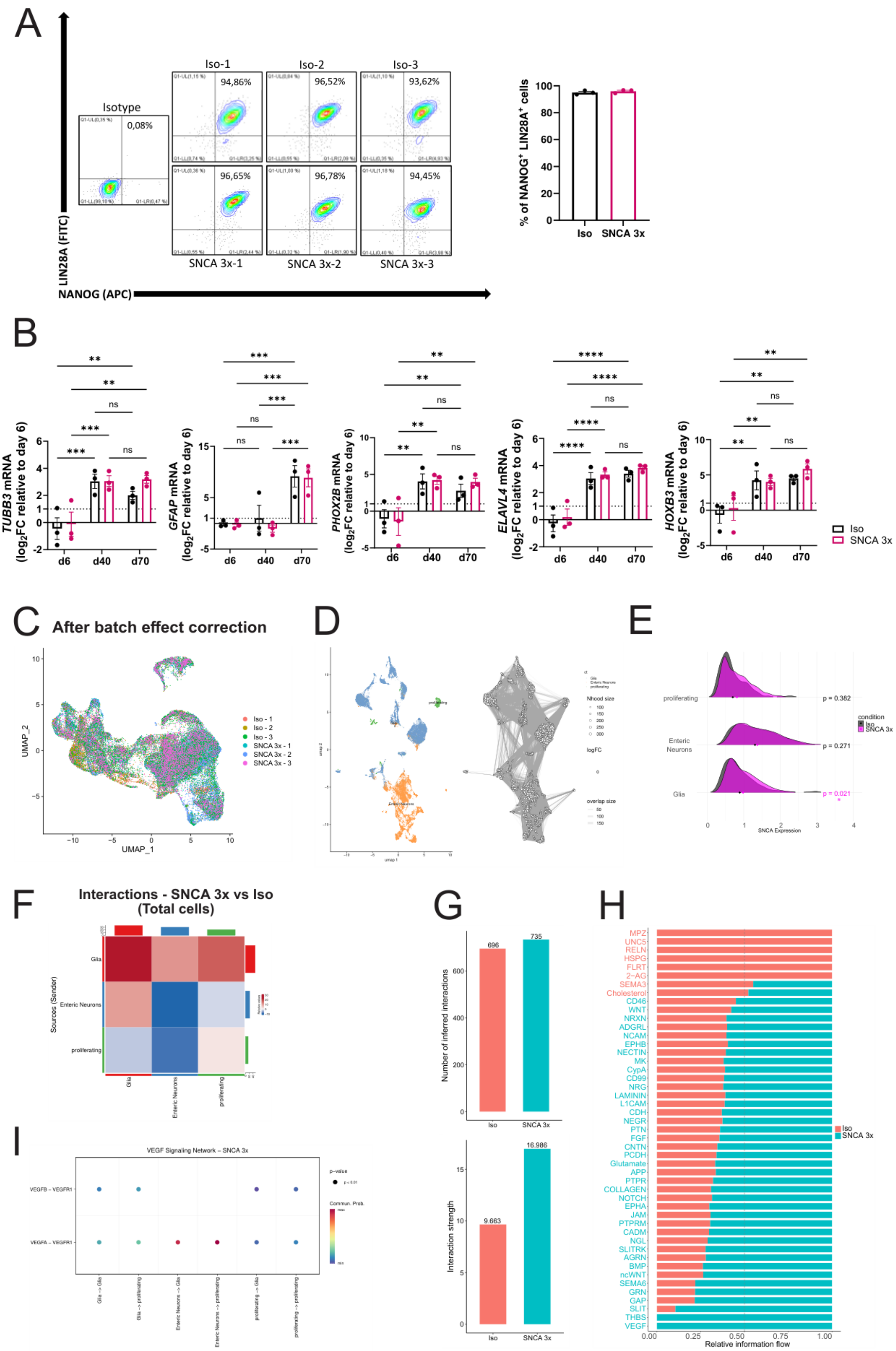

Figure S1. iPSC-ENLs as a model for studying synucleinopathies

(A) Flow cytometry of iPSC purity (NANOG+/LIN28A+) at differentiation day 0 (n=3 biological replicates/group). Mean ± SEM.

(B) RT-qPCR quantification of neuronal (*TUBB3*), glial (*GFAP*), and enteric (*PHOX2B*, *ELAVL4*, *HOXB3*) markers at days 6, 40, and 70. Log2 fold change relative to Iso (day 6). n=3 independent SNCA 3x and 3 isogenic lines per group, from two independent differentiations. Mean ± SEM, **$p < 0.01$, ***$p < 0.001$, ****$p < 0.0001$ (two-way ANOVA, Tukey's post-hoc).

(C) UMAP plot of scRNA-seq data from day 70 ENLs showing clustering after batch effect correction.

(D) Neighborhood graph of MiloR differential abundance testing in general clusters. Nodes (local cellular neighborhoods) are colored by log2FC (SNCA 3x/Iso) from depletion (red) to expansion (blue); non-differential neighborhoods are white. Node size reflects cell count; edges depict shared cells. Spatial FDR threshold = 0.2.

(E) Ridgeplot showing the expression of *SNCA* per condition considering each cluster identified. n=3 independent SNCA 3x and 3 isogenic lines per group, from one differentiation, p values calculated by unpaired two-tailed Student's t test.

(F) Heatmap comparing the cellular communication between SNCA 3x and Iso ENLs in total cells, with the top color bar representing the sum of the column values displayed in incoming signals and the right color bar representing the sum of outgoing signals, red or blue indicating increased or decreased signal of SNCA 3x compared with Iso, respectively. Data was generated using CellChat.

(G) Barplots showing the quantification of the number of inferred interactions (top) and interaction strength (bottom) in iPSC-ENLs total cells. Data was generated using CellChat.

(H) Differences in the overall signaling pathway between SNCA 3x and Iso ENLs in total cells, with the ranking indicating the importance of the pathways; red indicating the signaling pathways enriched in Iso, blue representing the signaling pathways enriched SNCA 3x, and black representing no difference in signaling pathway enrichment in groups.

(I) Bubble plot of VEGF signaling in total cells, showing the ligand-receptor pairs associated with this signaling pathways and the directionalities of the signals. Data was generated using CellChat.

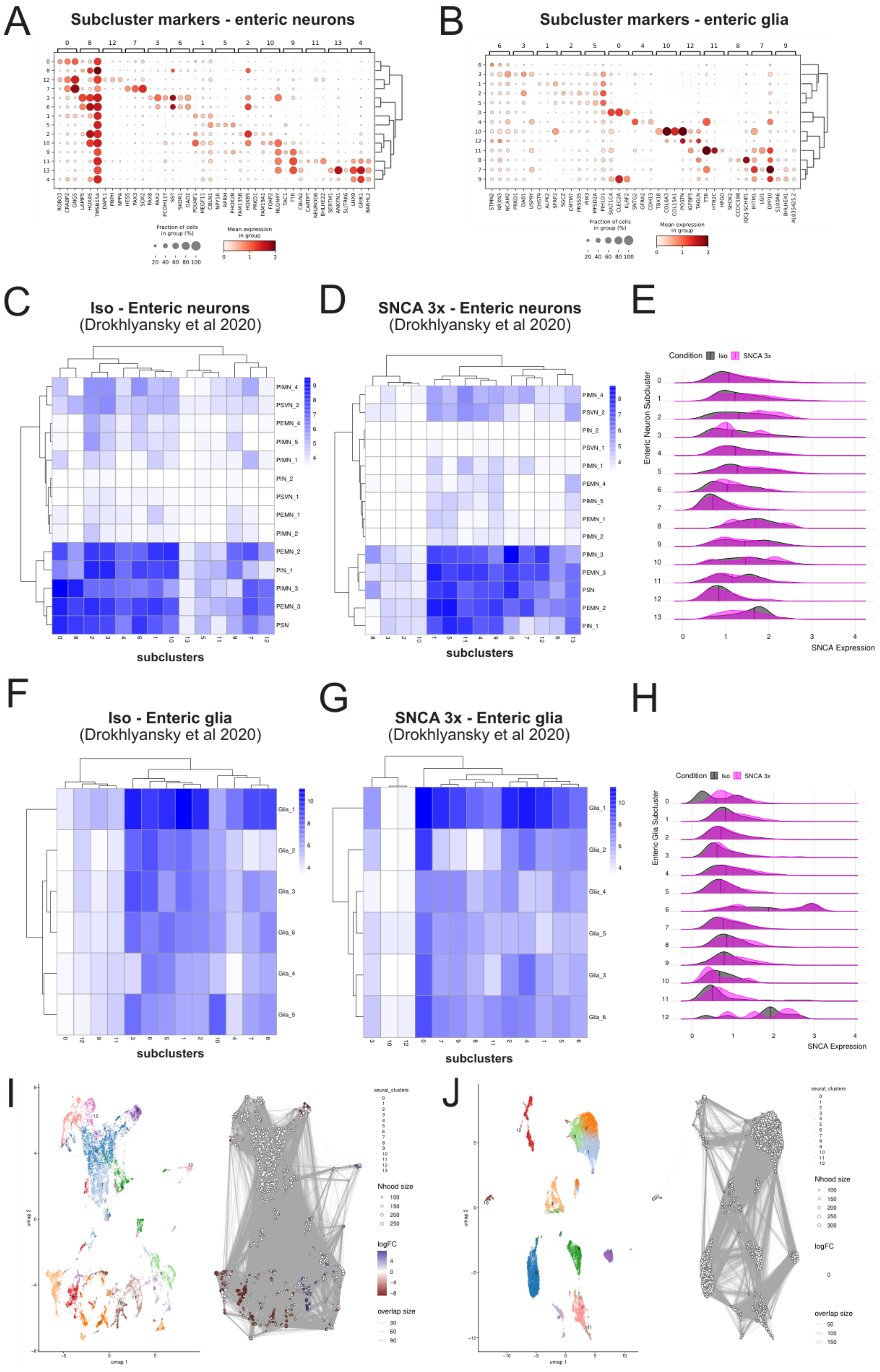
A
Subcluster markers - enteric neurons
B
Subcluster markers - enteric glia
Fraction of cells in group (%)
Mean expression in group
C
Iso - Enteric neurons
(Drokhlyansky et al 2020)
D
SNCA 3x - Enteric neurons
(Drokhlyansky et al 2020)
E
Condition Iso SNCA 3x
Enteric Neuron Subcluster
SNCA Expression
subclusters
F
Iso - Enteric glia
(Drokhlyansky et al 2020)
G
SNCA 3x - Enteric glia
(Drokhlyansky et al 2020)
H
Enteric Glia Subcluster
SNCA Expression
subclusters
I
J
seurat_clusters
Nhood size
logFC
overlap size
umap 1
umap 2

Figure S2. Subclustering matches distinct iPSC-ENL subpopulations to the human ENS

(A and B) Dotplots showing the top 3 subcluster markers for enteric neurons (A) and enteric glia (B), with clusterization based on the similarities between subclusters.

(C and D) Heatmaps showing sublcuster annotation similarities to the human ENS based on singleR scores. C and D represent Iso and SNCA 3x enteric neurons, respectively. Scores were calculated based on the populations depicted in Drokhlyansky et al 2020[27].

(E) Ridgeplot showing the expression levels of *SNCA* across the different enteric neuronal subclusters.

(F and G) Heatmaps showing sublcuster annotation similarities to the human ENS based on singleR scores. F and G represent Iso and SNCA 3x enteric glia, respectively. Scores were calculated based on the populations depicted in Drokhlyansky et al 2020. PEMN = putative excitatory motor neuron; PIMN = putative inhibitory motor neuron; PIN = putative interneurons; PSN = putative sensory neurons; PSVN = putative secretomotor/vasodilator neurons.

(H) Ridgeplot showing the expression levels of *SNCA* across the different enteric glial subclusters.

(I) Neighborhood graph of MiloR differential abundance testing in enteric neurons. Nodes (local cellular neighborhoods) are colored by log2FC (SNCA 3x/Iso) from depletion (red) to expansion (blue); non-differential neighborhoods are white. Node size reflects cell count; edges depict shared cells. Spatial FDR threshold = 0.2.

(J) Neighborhood graph of MiloR differential abundance testing in enteric glia. Nodes (local cellular neighborhoods) are colored by log2FC (SNCA 3x/Iso) from depletion (red) to expansion (blue); non-differential neighborhoods are white. Node size reflects cell count; edges depict shared cells. Spatial FDR threshold = 0.2.

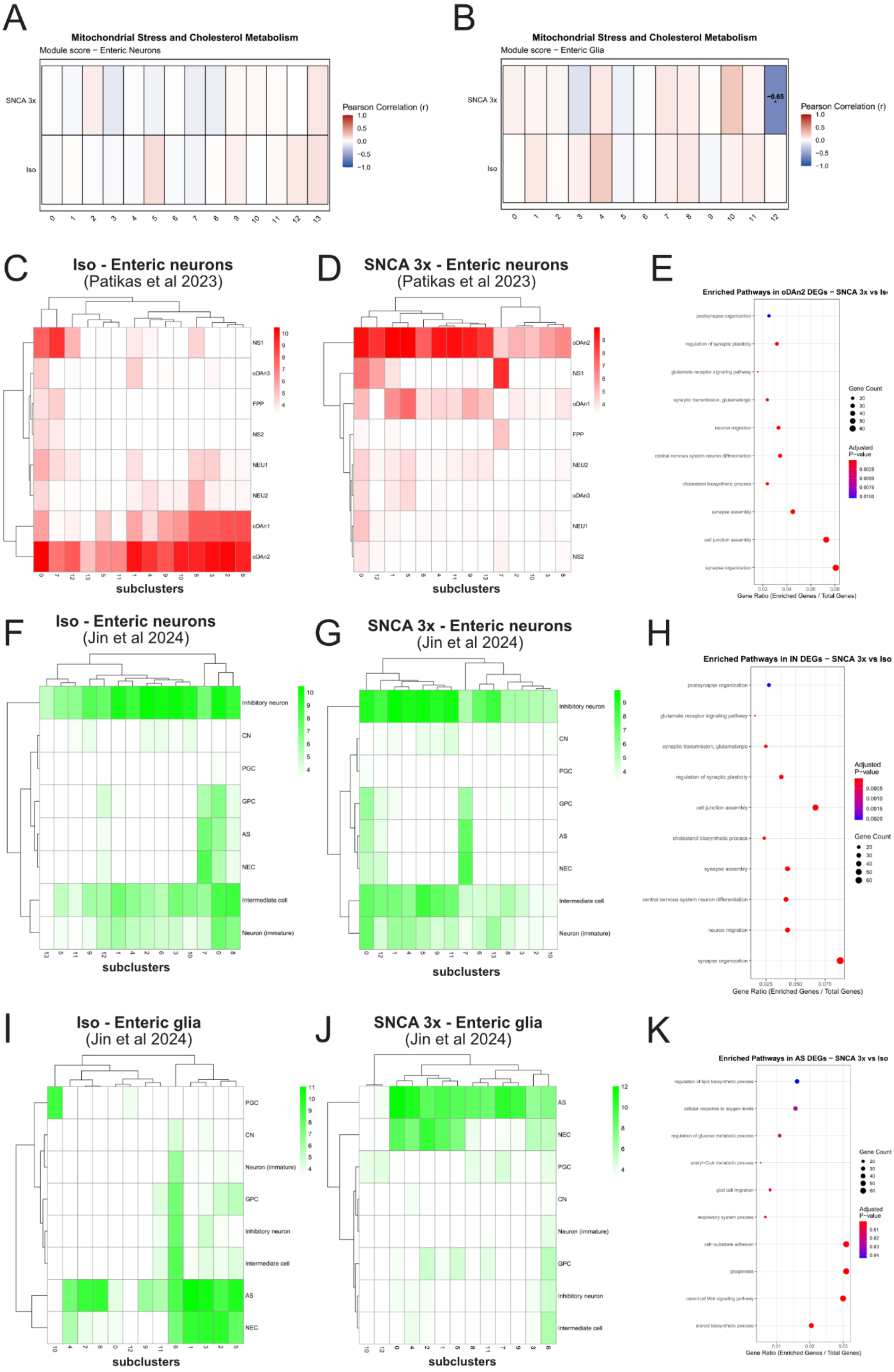

A
Mitochondrial Stress and Cholesterol Metabolism
Module score − Enteric Neurons
SNCA 3x
Iso
Pearson Correlation (r)
B
Mitochondrial Stress and Cholesterol Metabolism
Module score − Enteric Glia
−0.65
Pearson Correlation (r)
C
Iso - Enteric neurons
(Patikas et al 2023)
NS1
oDAn3
FPP
NS2
NEU1
NEU2
oDAn1
oDAn2
subclusters
D
SNCA 3x - Enteric neurons
(Patikas et al 2023)
subclusters
E
Enriched Pathways in oDAn2 DEGs − SNCA 3x vs Iso
postsynapse organization
regulation of synaptic plasticity
glutamate receptor signaling pathway
synaptic transmission, glutamatergic
neuron migration
central nervous system neuron differentiation
cholesterol biosynthetic process
synapse assembly
cell junction assembly
synapse organization
Gene Count
Adjusted P−value
Gene Ratio (Enriched Genes / Total Genes)
F
Iso - Enteric neurons
(Jin et al 2024)
Inhibitory neuron
CN
PGC
GPC
AS
NEC
Intermediate cell
Neuron (immature)
subclusters
G
SNCA 3x - Enteric neurons
(Jin et al 2024)
subclusters
H
Enriched Pathways in IN DEGs − SNCA 3x vs Iso
postsynapse organization
glutamate receptor signaling pathway
synaptic transmission, glutamatergic
regulation of synaptic plasticity
cell junction assembly
cholesterol biosynthetic process
synapse assembly
central nervous system neuron differentiation
neuron migration
synapse organization
Adjusted P−value
Gene Count
Gene Ratio (Enriched Genes / Total Genes)
I
Iso - Enteric glia
(Jin et al 2024)
subclusters
J
SNCA 3x - Enteric glia
(Jin et al 2024)
subclusters
K
Enriched Pathways in AS DEGs − SNCA 3x vs Iso
regulation of lipid biosynthetic process
cellular response to oxygen levels
regulation of glucose metabolic process
acetyl−CoA metabolic process
glial cell migration
respiratory system process
cell−substrate adhesion
gliogenesis
canonical Wnt signaling pathway
steroid biosynthetic process
Gene Count
Adjusted P−value
Gene Ratio (Enriched Genes / Total Genes)

Figure S3. SNCA 3x ENLs share signatures with cortical and midbrain organoids

(A and B) Heatmaps showing the correlation between module scores for mitochondrial stress and cholesterol metabolism in enteric neurons (A) and enteric glia (B). A threshold of absolute correlation >0.5 and p<0.05 was applied to all samples.

(C and D) Heatmaps showing sublcuster annotation similarities to the human ENS based on singleR scores. C and D represent Iso and SNCA 3x enteric neurons, respectively. Scores were calculated based on the populations depicted in Patikas et al. 2023. oDAn = organoid dopaminergic neuron; NEU = non-dopaminergic neurons; FPP = floor plate progenitors; NS = neural stem cell.

(E) Integrated enrichment including Reactome, KEGG and Gene Ontology Biological Processes, Molecular Function and Cellular Component based on the differentially expressed genes between SNCA 3x and Iso when enteric neurons were subsetted with the organoid dopaminergic 2 (oDAn2) annotation.

(F and G) Heatmaps showing sublcuster annotation similarities to the human ENS based on singleR scores. F and G represent Iso and SNCA 3x enteric neurons, respectively. Scores were calculated based on the populations depicted in Jin et al. 2024. NEC = neural ectoderm cells; AS = astrocyte; CN = cortical neuron; PGC = progenitor cells; GPC = glial precursor cells.

(H) Integrated enrichment including Reactome, KEGG and Gene Ontology Biological Processes, Molecular Function and Cellular Component based on the differentially expressed genes between SNCA 3x and Iso when enteric neurons were subsetted with the inhibitory neuron annotation.

(I and J) Heatmaps showing sublcuster annotation similarities to the human ENS based on singleR scores. F and G represent Iso and SNCA 3x enteric glia, respectively. Scores were calculated based on the populations depicted in Jin et al. 2024.

(K) Integrated enrichment including Reactome, KEGG and Gene Ontology Biological Processes, Molecular Function and Cellular Component based on the differentially expressed genes between SNCA 3x and Iso when enteric glia were subsetted with the astrocyte (AS) annotation.

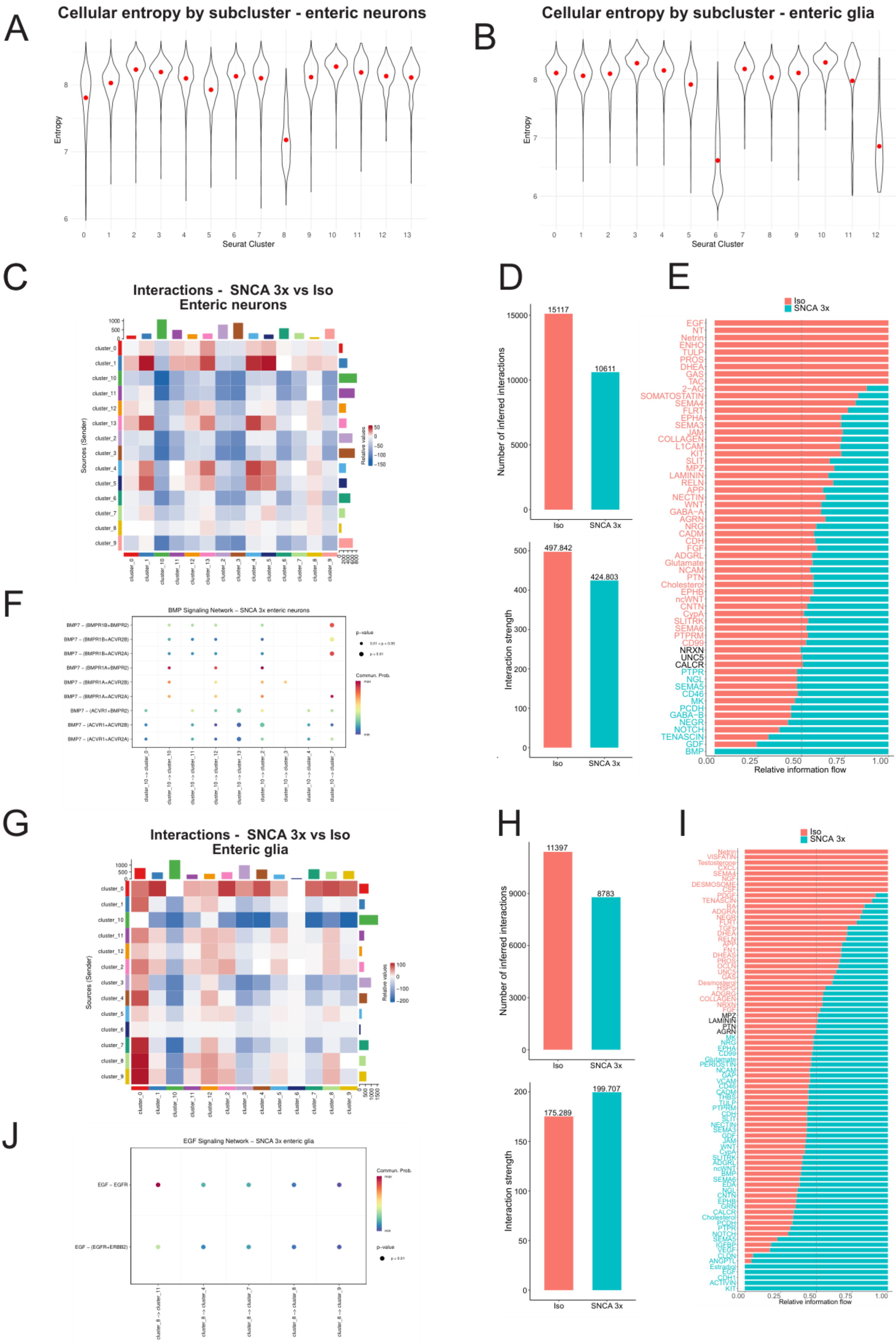
A
Cellular entropy by subcluster - enteric neurons
Entropy
Seurat Cluster
B
Cellular entropy by subcluster - enteric glia
Entropy
Seurat Cluster
C
Interactions - SNCA 3x vs Iso
Enteric neurons
Sources (Sender)
Relative values
D
Number of inferred interactions
15117
10611
Iso
SNCA 3x
Interaction strength
497.842
424.803
E
Iso
SNCA 3x
Relative information flow
F
BMP Signaling Network – SNCA 3x enteric neurons
p-value
Commun. Prob.
G
Interactions - SNCA 3x vs Iso
Enteric glia
Sources (Sender)
Relative values
H
Number of inferred interactions
11397
8783
Iso
SNCA 3x
Interaction strength
175.289
199.707
I
Iso
SNCA 3x
Relative information flow
J
EGF Signaling Network – SNCA 3x enteric glia
EGF - EGFR
EGF - (EGFR+ERBB2)
Commun. Prob.
p-value

Figure S4. SNCA 3x drives basal mitochondrial dysfunction and alters enteric neuron-glia communication in iPSC-ENLs

(A and B) Calculation of cellular entropy by subcluster using TSCAN for enteric neurons (A) and enteric glial cells (B).

(C) Heatmap of cellular communication in enteric neuron subclusters, with the top color bar representing the sum of the column values displayed in incoming signals and the right color bar representing the sum of outgoing signals, red or blue indicating increased or decreased signal of SNCA 3x compared with Iso, respectively. Data was generated using CellChat.

(D) Barplots showing the quantification of the number of inferred interactions (top) and interaction strength (bottom) in enteric neuron subclusters. Data was generated using CellChat.

(E) Differences in the overall signaling pathway between SNCA 3x and Iso ENLs in enteric neuron subclusters, with the ranking indicating the importance of the pathways; red = pathways enriched in Iso, blue = pathways enriched in SNCA 3x, black = no difference in signaling pathway enrichment in groups.

(F) Bubble plot of BMP signaling in enteric neurons, showing the ligand-receptor pairs associated with this signaling pathways and the directionalities of the signals. Data was generated using CellChat.

(G) Heatmap comparing the cellular communication between SNCA 3x and Iso ENLs in enteric glia subclusters, with the top color bar representing the sum of the column values displayed in incoming signals and the right color bar representing the sum of outgoing signals, red or blue indicating increased or decreased signal of SNCA 3x compared with Iso, respectively. Data was generated using CellChat.

(H) Barplots showing the quantification of the number of inferred interactions (top) and interaction strength (bottom) in enteric glia subclusters. Data was generated using CellChat.

(I) Differences in the overall signaling pathway between SNCA 3x and Iso ENLs in enteric glia subclusters, with the ranking indicating the importance of the pathways; red = pathways enriched in Iso, blue = pathways enriched in SNCA 3x, black = no difference in signaling pathway enrichment in groups

(J) Bubble plot of EGF signaling in enteric glia, showing the ligand-receptor pairs associated with this signaling pathways and the directionalities of the signals. Data was generated using CellChat.

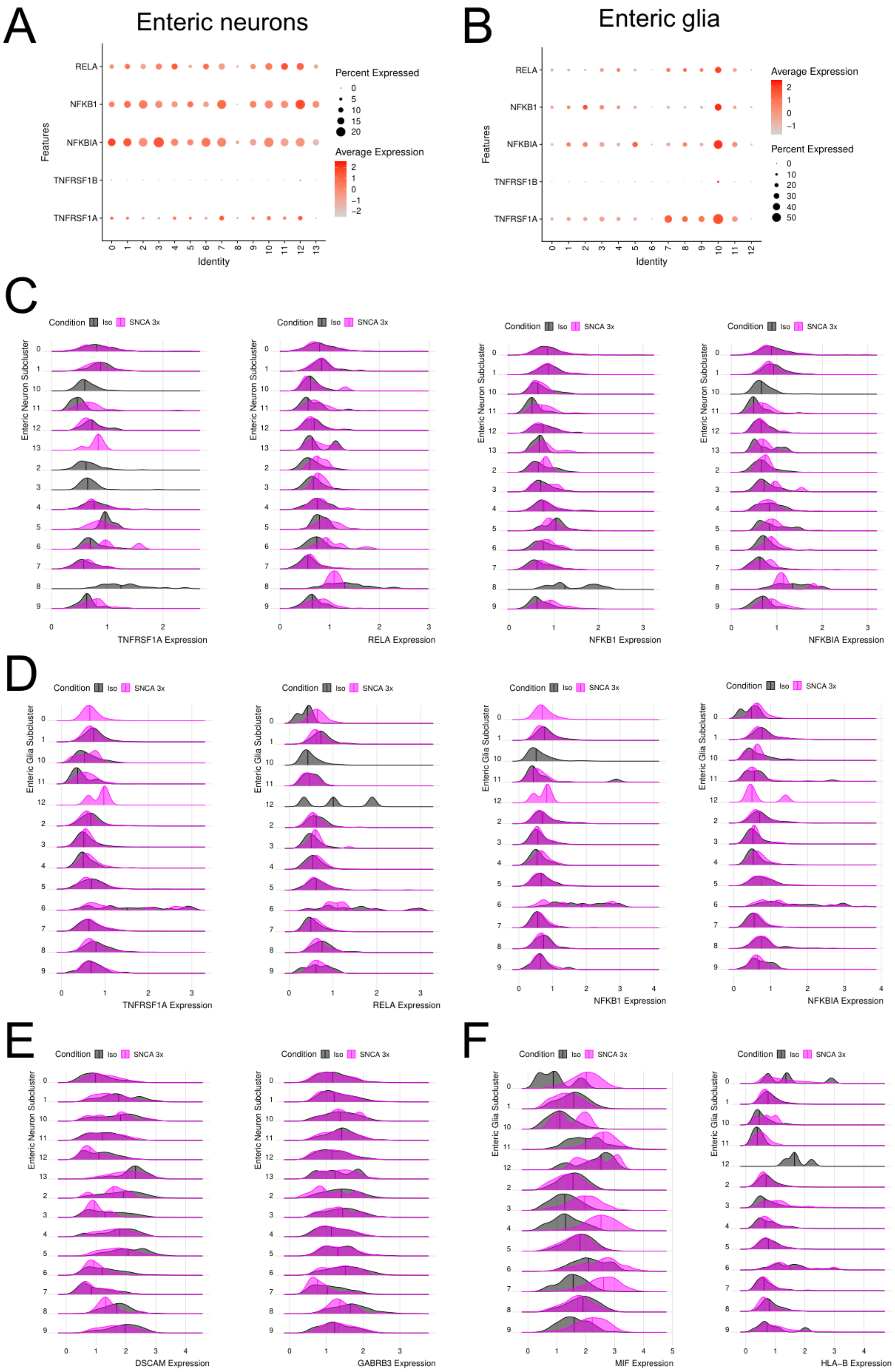
A
Enteric neurons
RELA
NFKB1
NFKBIA
TNFRSF1B
TNFRSF1A
Features
Identity
Percent Expressed
Average Expression
B
Enteric glia
C
Condition
Iso
SNCA 3x
Enteric Neuron Subcluster
TNFRSF1A Expression
RELA Expression
NFKB1 Expression
NFKBIA Expression
D
Enteric Glia Subcluster
E
DSCAM Expression
GABRB3 Expression
F
MIF Expression
HLA–B Expression

Figure S5. SNCA 3x induces cell-type-specific pathological priming under basal conditions, confirmed by differential expression of TNF pathway and synaptic markers

(A and B) Dotplots showing the average and percentage of expression of TNF pathway genes for enteric neuron **(A)** and glial **(B)** subclusters.

(C) Ridgeplots showing the expression of TNF pathway genes (*TNFRSF1A*, *RELA*, *NFKB1* and *NFKBIA*) per condition in enteric neuron subclusters. n=3 independent SNCA 3x and 3 isogenic lines per group, from one differentiation.

(D) Ridgeplots showing the expression of TNF pathway genes (*TNFRSF1A*, *RELA*, *NFKB1* and *NFKBIA*) per condition in enteric glia subclusters. n=3 independent SNCA 3x and 3 isogenic lines per group, from one differentiation.

(E) Ridgeplots showing the expression of synapse related genes (*DSCAM* and *GABRB3*) per condition in enteric neuron subclusters. n=3 independent SNCA 3x and 3 isogenic lines per group, from one differentiation.

(F) Ridgeplots showing the expression of inflammation related genes (*MIF* and *HLA-B*) per condition in enteric glia subclusters. n=3 independent SNCA 3x and 3 isogenic lines per group, from one differentiation.

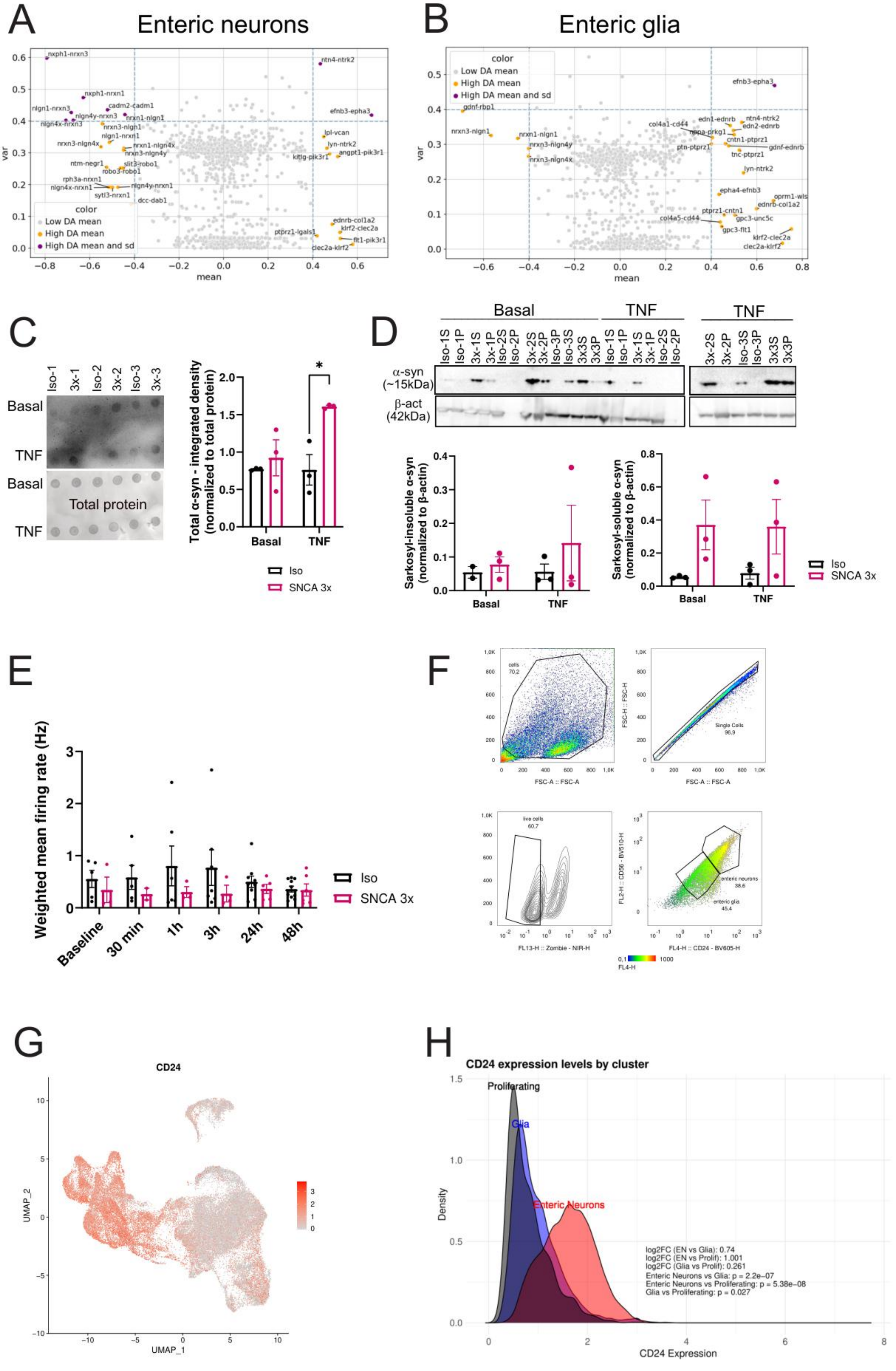
A
Enteric neurons
color
Low DA mean
High DA mean
High DA mean and sd
nxph1-nrxn3
nxph1-nrxn1
nlgn1-nrxn3
cadm2-cadm1
nlgn4y-nrxn3
nrxn1-nlgn1
nlgn4x-nrxn3
nrxn3-nlgn1
nlgn1-nrxn1
nrxn3-nlgn4x
nrxn1-nlgn4x
nrxn3-nlgn4y
ntm-negr1
slit3-robo1
robo3-robo1
rph3a-nrxn1
nlgn4x-nrxn1
nlgn4y-nrxn1
sytl3-nrxn1
dcc-dab1
ntn4-ntrk2
efnb3-epha3
lpl-vcan
lyn-ntrk2
kitlg-pik3r1
angpt1-pik3r1
ednrb-col1a2
klrf2-clec2a
ptprz1-lgals1
flt1-pik3r1
clec2a-klrf2
var
mean
B
Enteric glia
color
Low DA mean
High DA mean
High DA mean and sd
efnb3-epha3
gdnf-rbp1
nrxn3-nlgn1
nrxn1-nlgn1
nrxn3-nlgn4y
nrxn3-nlgn4x
col4a1-cd44
nppa-prkg1
ptn-ptprz1
edn1-ednrb
ntn4-ntrk2
edn2-ednrb
cntn1-ptprz1
gdnf-ednrb
tnc-ptprz1
lyn-ntrk2
epha4-efnb3
oprm1-wls
ednrb-col1a2
ptprz1-cntn1
col4a5-cd44
gpc3-unc5c
gpc3-flt1
klrf2-clec2a
clec2a-klrf2
var
mean
C
Iso-1
3x-1
Iso-2
3x-2
Iso-3
3x-3
Basal
TNF
Basal
Total protein
TNF
Total α-syn - integrated density (normalized to total protein)
Basal
TNF
Iso
SNCA 3x
D
Basal
TNF
TNF
Iso-1S
Iso-1P
3x-1S
3x-1P
Iso-2S
Iso-2P
3x-2S
3x-2P
Iso-3P
Iso-3S
3x3S
3x3P
α-syn (~15kDa)
β-act (42kDa)
Sarkosyl-insoluble α-syn (normalized to β-actin)
Sarkosyl-soluble α-syn (normalized to β-actin)
Basal
TNF
Iso
SNCA 3x
E
Weighted mean firing rate (Hz)
Baseline
30 min
1h
3h
24h
48h
Iso
SNCA 3x
F
cells
70,2
FSC-A :: FSC-A
FSC-H :: FSC-H
Single Cells
96,9
live cells
60,7
FL13-H :: Zombie - NIR-H
FL2-H :: CD56 - BV510-H
enteric neurons
38,6
enteric glia
45,4
FL4-H :: CD24 - BV605-H
FL4-H
G
CD24
UMAP_2
UMAP_1
H
CD24 expression levels by cluster
Proliferating
Glia
Enteric Neurons
Density
CD24 Expression
log2FC (EN vs Glia): 0.74
log2FC (EN vs Prolif): 1.001
log2FC (Glia vs Prolif): 0.261
Enteric Neurons vs Glia: p = 2.2e−07
Enteric Neurons vs Proliferating: p = 5.38e−08
Glia vs Proliferating: p = 0.027

Figure S6. TNF uncovers genotype-specific α-Syn accumulation and synaptic dysfunction in SNCA 3x ENLs

(A and B) Ligand-Receptor pair analysis of the scRNAseq data in enteric neurons (A) and enteric glia (B). These plots depict pairs that show both a large mean change as well a large variance in the population (purple). Cells that don't make the variance cut-off of 0.3 are shown in orange. Cells that don't make the mean cut-off of 0.3 are shown in grey.

(C) Dot blot analysis of total α-syn (Syn-1 antibody, BD Biosciences, cat# 610787) quantified intracellularly in iPSC-ENLs, n=3 independent SNCA 3x and 3 isogenic lines per group, from one differentiation, mean ± SEM, *p<0.05 by two-way ANOVA with Sidak post-hoc. Basal refers to cells treated with vehicle used to dilute the TNF (DPBS+0.1% BSA).

(D) Sarkosyl-aggregation assay of α-syn blot (2A7 antibody, Novus, Cat#NBP1-05194) and quantification in IPSC-ENLs. Data was normalized to b-actin. S=supernatant, P=pellet. n=3 independent SNCA 3x and 3 isogenic lines per group, from one differentiation, mean ± SEM. Basal refers to cells treated with vehicle used to dilute the TNF (DPBS+0.1% BSA).

(E) Quantification of the weighted mean firing rate generated from the MEA data, n=wells of a CytoView MEA 48-well plate, representative of n=3 independent SNCA 3x and 3 isogenic lines per group, from two independent differentiations, mean ± SEM.

(F) Gating strategy used to generate the flow cytometry data used in the paper.

(G) UMAP plot obtained from scRNA-seq analysis of Iso and SNCA 3x ENLs at day 70 after start of differentiation, showing *CD24* expression.

(H) Ridgeplot obtained from scRNA-seq analysis of Iso and SNCA 3x ENLs at day 70 after start of differentiation, comparing the CD24 expression per cluster, showing log2FC and p-values calculated by unpaired two-tailed Student's t test.

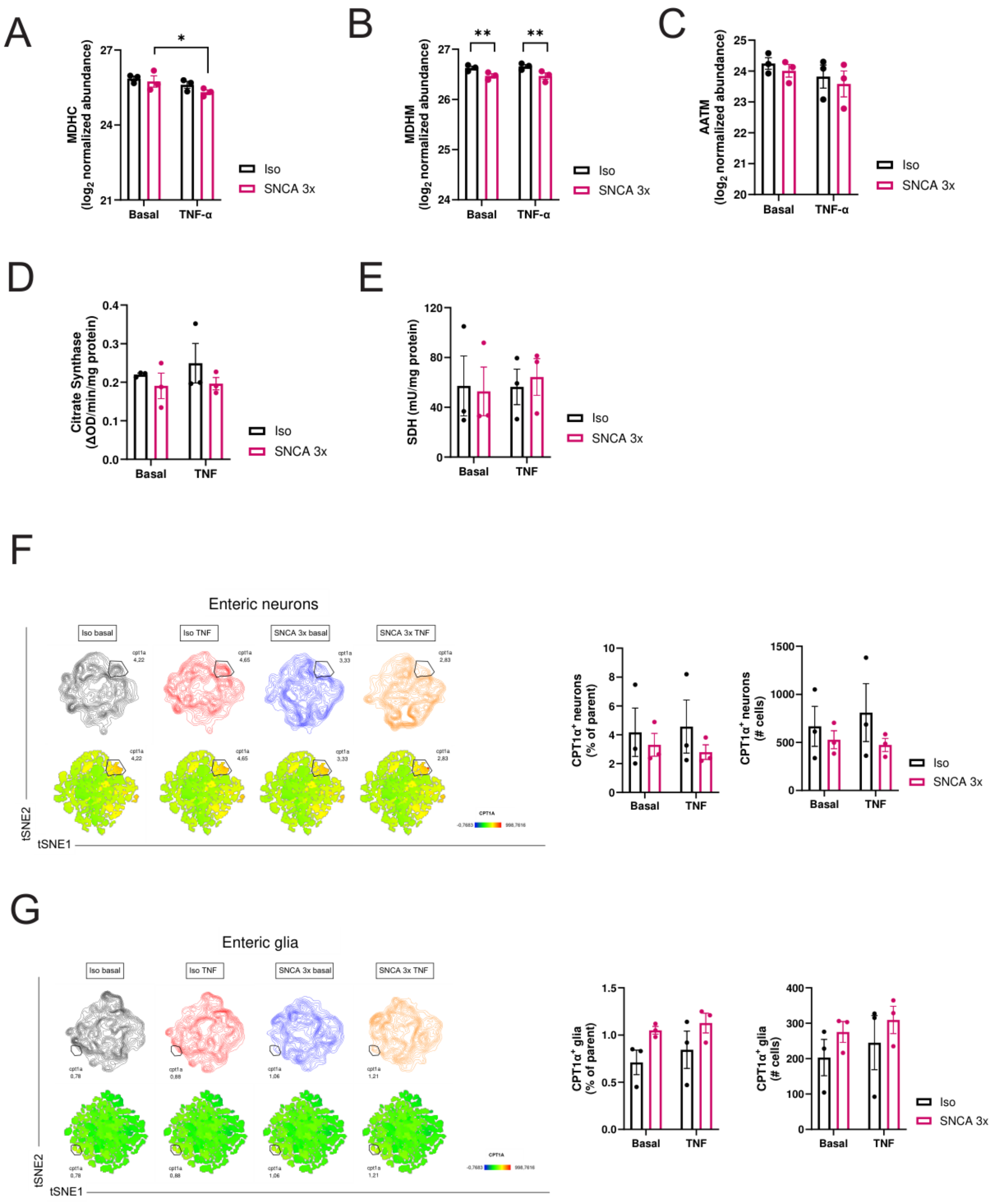

Figure S7. Validation of MAS, TCA and FAO dysfunction in iPSC-ENLs

(A-C) Quantification of cytosolic malate dehydrogenase (MDHC) (A), mitochondrial malate dehydrogenase (MDHM) (B) and mitochondrial aspartate aminotransferase (AATM) (C) from the proteomics data. n=3 independent SNCA 3x and 3 isogenic lines per group, from one differentiation, mean ± SEM, *p<0.05. Raw p-values were calculated using a linear model and empirical Bayes moderation (limma). Basal refers to cells treated with vehicle used to dilute the TNF (DPBS+0.1% BSA).

(D and E) Quantification of Citrate Synthase (D) and Succinate Dehydrogenase (E) enzymatic activities using specific kits (ab119692 and ab228560, respectively, both from Abcam). n=3 independent SNCA 3x and 3 isogenic lines per group, from two differentiations, mean ± SEM.

(F and G) Concatenated t-SNE plots from flow cytometry data of all lines highlighting CPT1α^{high} populations across genotypes and conditions and quantification of the percentage and total CPT1α^{high} cells in enteric neurons (CD56^{+}CD24^{high}) (F) and in enteric glia (CD56^{+}CD24^{low}) (G). n=3 independent SNCA 3x and 3 isogenic lines per group, from two independent differentiations, mean ± SEM.

Source Data

Uncropped scans of blots within Supplementary Figures

Fig S6C

Uncropped dotblots (left, α-synuclein; right, total protein)

Upper row: Basal samples

Lower row: TNF stimulated samples

Order of samples (from left to right, for both blots): Iso-1, 3x-1, Iso-2, 3x-2, Iso-3, 3x-3

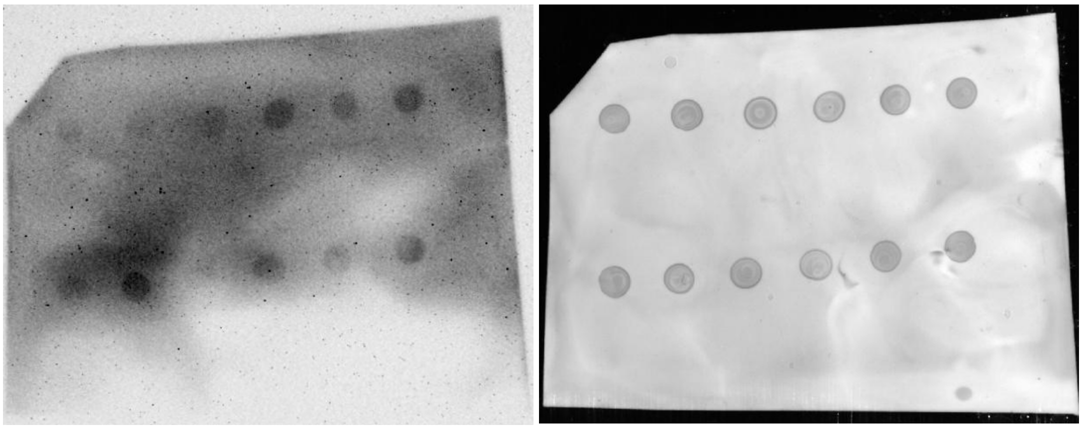

Fig S6D

Uncropped blots (α-synuclein)

Left: first membrane. Order of the samples (left to right, Basal samples: Iso-1S, Iso-1P, 3x-1S, 3x-1P, Iso-2S, Iso-2P, 3x-2S, 3x-2P, Iso-3P, Iso-3S, 3x-3S, 3x-3P; TNF-treated samples: Iso-1S, Iso-1P, 3x-1P, Iso-2S, Iso-2P)

Right: second membrane. Order of the samples: (left to right, TNF-treated samples: 3x-2S, 3x-2P, Iso-3S, Iso-3P, 3x-3S, 3x-3P

S= supernatant, P=pellet

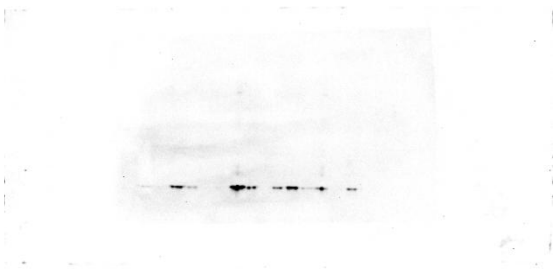

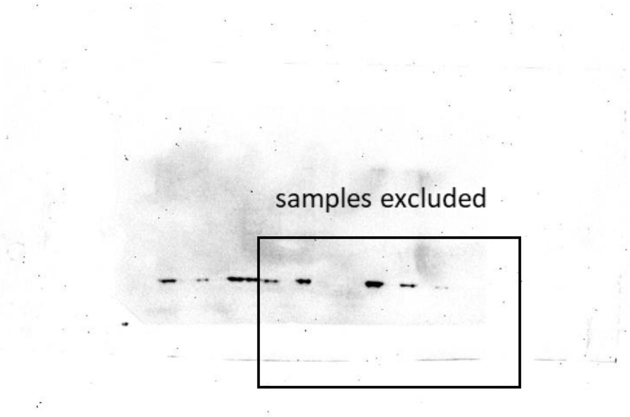

Uncropped blots (β-actin)

<u>Left:</u> first membrane. Order of the samples (left to right, Basal samples: Iso-1S, Iso-1P, 3x-1S, 3x-1P, Iso-2S, Iso-2P, 3x-2S, 3x-2P, Iso-3P, Iso-3S, 3x-3S, 3x-3P; TNF-treated samples: Iso-1S, Iso-1P, 3x-1P, Iso-2S, Iso-2P)

<u>Right:</u> second membrane. Order of the samples: (left to right, TNF-treated samples: 3x-2S, 3x-2P, Iso-3S, Iso-3P, 3x-3S, 3x-3P

S= supernatant, P=pellet

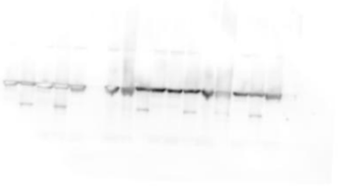

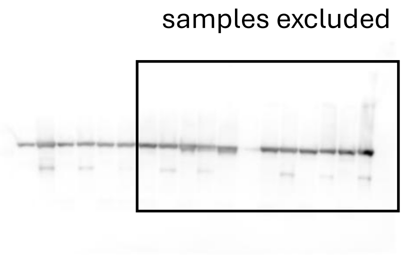

Supplement: Supplementary file 1 — Supplementary Information [file 41467_2026_71317_MOESM1_ESM.pdf]
