# Supplementary material for: TNF alpha unmasks enteric malate aspartate shuttle dysfunction bridging Parkinson disease and intestinal inflammation
Source: Nat Commun. 2026 Apr 1;17:3217. doi: 10.1038/s41467-026-71317-y (PMC13057073; doi:10.1038/s41467-026-71317-y)
Supplement: Supplementary file 2 — Description of Additional Supplementary Files [file 41467_2026_71317_MOESM2_ESM.pdf]

Description of additional supplementary files

File name: Supplementary Data 1

Description: Primer sequences used in this study

File name: Supplementary Data 2

Description: Subcluster markers annotation

File name: Supplementary Data 3

Description: SingleR annotation scores

File name: Supplementary Data 4

Description: Differentially expressed genes between conditions

File name: Supplementary Data 5

Description: MiloR analysis

File name: Supplementary Data 6

Description: Integrated enrichment analysis

File name: Supplementary Data 7

Description: CellChat analysis results

File name: Supplementary Data 8

Description: Pre-processed proteomics data and differentially expressed proteins (DEPs)

File name: Supplementary Data 9

Description: Molecular function enrichment of proteomics data

File name: Supplementary Data 10

Description: Pre-processed metabolomics data

File name: Supplementary Data 11

Description: Metadata for the Ulcerative Colitis patient cohort used
