# Supplementary material for: TNF alpha unmasks enteric malate aspartate shuttle dysfunction bridging Parkinson disease and intestinal inflammation
Source: Nat Commun. 2026 Apr 1;17:3217. doi: 10.1038/s41467-026-71317-y (PMC13057073; doi:10.1038/s41467-026-71317-y)
Supplement: Supplementary file 14 — Reporting Summary [file 41467_2026_71317_MOESM14_ESM.pdf]

nature portfolio

Corresponding author(s): Beate Winner

Last updated by author(s): Mar 3, 2026

Reporting Summary

Nature Portfolio wishes to improve the reproducibility of the work that we publish. This form provides structure for consistency and transparency in reporting. For further information on Nature Portfolio policies, see our Editorial Policies and the Editorial Policy Checklist.

Statistics

For all statistical analyses, confirm that the following items are present in the figure legend, table legend, main text, or Methods section.

n/a	Confirmed	
☐	☒	The exact sample size (n) for each experimental group/condition, given as a discrete number and unit of measurement
☐	☒	A statement on whether measurements were taken from distinct samples or whether the same sample was measured repeatedly
☐	☒	The statistical test(s) used AND whether they are one- or two-sided<br>*Only common tests should be described solely by name; describe more complex techniques in the Methods section.*
☐	☒	A description of all covariates tested
☐	☒	A description of any assumptions or corrections, such as tests of normality and adjustment for multiple comparisons
☐	☒	A full description of the statistical parameters including central tendency (e.g. means) or other basic estimates (e.g. regression coefficient) AND variation (e.g. standard deviation) or associated estimates of uncertainty (e.g. confidence intervals)
☐	☒	For null hypothesis testing, the test statistic (e.g. F, t, r) with confidence intervals, effect sizes, degrees of freedom and P value noted<br>*Give P values as exact values whenever suitable.*
☒	☐	For Bayesian analysis, information on the choice of priors and Markov chain Monte Carlo settings
☒	☐	For hierarchical and complex designs, identification of the appropriate level for tests and full reporting of outcomes
☐	☒	Estimates of effect sizes (e.g. Cohen's d, Pearson's r), indicating how they were calculated

Our web collection on statistics for biologists contains articles on many of the points above.

Software and code

Policy information about availability of computer code

Data collection	Seahorse Wave Desktop 2.6.3, Agilent, https://www.agilent.com/en/product/cell-analysis/real-time-cell-metabolic-analysis/xf-software/seahorse-wave-desktop-software-740897<br>CytExpert 2.4, Beckman Coulter, https://www.beckman.de/flow-cytometry/research-flow-cytometers/cytoflex/software<br>LightCycler 480 1.5.0, Roche, https://lifescience.roche.com/global/en/products/product-category/lightcycler.html#4<br>iBright system 5.3.0, Thermo Fisher, https://www.thermofisher.com/de/en/home/life-science/protein-biology/protein-assays-analysis/western-blotting/detect-proteins-western-blot/western-blot-imaging-analysis/ibright-systems/software.html<br>Image Lab 3.0, Biorad, https://www.bio-rad.com/de-de/product/image-lab-software<br>AxIS 2.5.2, Axion Biosystems, https://www.axionbiosystems.com/products/mea/mea-software<br>cellSens 4.4, Evident Scientific, https://evidentscientific.com/en/downloads?product=cellSens<br>Zen 3.3, Zeiss, https://www.zeiss.com/microscopy/en/products/software/zeiss-zen.html<br>SkanIT 7.0.2, Thermo Fisher, https://www.thermofisher.com/de/de/home/life-science/lab-equipment/microplate-instruments/plate-readers/software.html<br>NovaSeq Control Software 1.7, Illumina, https://emea.support.illumina.com/downloads/novaseq-control-software-v1-7.html<br>bcl2fastq 2.20, Illumina, https://emea.support.illumina.com/sequencing/sequencing_software/bcl2fastq-conversion-software.html<br>CellRanger 7.1.0, 10x Genomics, https://www.10xgenomics.com/support/software/cell-ranger/7<br>Fragpipe 23.0, Nesvilab, https://fragpipe.nesvilab.org/<br>Compound Discoverer 3.3, Thermo Fisher, https://www.thermofisher.com/de/de/home/industrial/mass-spectrometry/liquid-chromatography-mass-spectrometry-lc-ms/lc-ms-software/multi-omics-data-analysis/compound-discoverer-software.html

Data analysis

Graph Pad Prism 10, Graph Pad, https://www.graphpad.com
Seahorse Wave Desktop 2.6.3, Agilent, https://www.agilent.com/en/product/cell-analysis/real-time-cell-metabolic-analysis/xf-software/seahorse-wave-desktop-software-740897
CytExpert 2.4, Beckman Coulter, https://www.beckman.de/flow-cytometry/research-flow-cytometers/cytoflex/software
FIJI 2.14.0, ImageJ, https://imagej.net/software/fiji/downloads,
LightCycler 480 1.5.0, Roche, https://lifescience.roche.com/global/en/products/product-category/lightcycler.html#4
AxIS Metric Plotting, Axion Biosystems, https://www.axionbiosystems.com/products/mea/mea-software
Cell Profiler, 4.2.1, Broad Institute, https://cellprofiler.org/
R 4.3.3, Posit, https://posit.co/download/rstudio-desktop/
Seurat 5.1.0, Satija lab, https://satijalab.org/seurat/
Compound Discoverer 3.3, Thermo Fisher, https://www.thermofisher.com/de/de/home/industrial/mass-spectrometry/liquid-chromatography-mass-spectrometry-lc-ms/lc-ms-software/multi-omics-data-analysis/compound-discoverer-software.html
MetaboAnalyst 6.0, Wishart group, University of Alberta, https://www.metaboanalyst.ca/
Ingenuity Pathway Analysis 23.0, QIAGEN, https://digitalinsights.qiagen.com/products-overview/discovery-insights-portfolio/analysis-and-visualization/qiagen-ipa/
SingleR 2.10.0, Aran et al 2019, https://www.bioconductor.org/packages/release/bioc/html/SingleR.html
Scanpy 1.11, Scanpy, https://scanpy.readthedocs.io/en/stable/release-notes/index.html
CellChat 2.1.2, Jin et al 2021, https://github.com/sqjin/CellChat
Slingshot 2.10.0, Street et al. 2018, https://bioconductor.org/packages/release/bioc/html/slingshot.html
scProportionTest 0.0.0.9000, Miller et al. 2021, https://github.com/rpolicastro/scProportionTest
limma 3.65.1, Bioconductor, https://bioconductor.org/packages/devel/bioc/html/limma.html
FlowJo 10.1, BD Biosciences, https://www.flowjo.com/

For manuscripts utilizing custom algorithms or software that are central to the research but not yet described in published literature, software must be made available to editors and reviewers. We strongly encourage code deposition in a community repository (e.g. GitHub). See the Nature Portfolio guidelines for submitting code & software for further information.

Data

Policy information about availability of data

All manuscripts must include a data availability statement. This statement should provide the following information, where applicable:

- Accession codes, unique identifiers, or web links for publicly available datasets
- A description of any restrictions on data availability
- For clinical datasets or third party data, please ensure that the statement adheres to our policy

The scRNAseq data generated in this study have been deposited in the GEO database under accession code GSE301050 [https://www.ncbi.nlm.nih.gov/geo/query/acc.cgi?acc=GSE301050]. The proteomics data generated in this study have been deposited in the ProteomeXChange database under accession code PXD075048 [https://proteomecentral.proteomexchange.org/cgi/GetDataset?ID=PXD075048] and in the MassIVE database under accession code MSV000101003 [https://massive.ucsd.edu/ProteoSAFe/dataset.jsp?task=b41f61176673413694a7cf65448de9fe]. The metabolomics data generated in this study have been deposited in the MassIVE database under accession code MSV000098366 [https://massive.ucsd.edu/ProteoSAFe/dataset.jsp?task=85492deedc7c40a39a3568287f5d398b]. Source data are provided as a Source Data file with this paper

All codes used in this publication, together with the Seurat object used to generate all our scRNAseq data are available at [Ghirotto, Bruno (2025), "ENS alpha synuclein paper 2025", Mendeley Data, V2, [https://data.mendeley.com/datasets/v8dknj466y/2].

Research involving human participants, their data, or biological material

Policy information about studies with human participants or human data. See also policy information about sex, gender (identity/presentation), and sexual orientation and race, ethnicity and racism.

Reporting on sex and gender

This study utilized IPSC from both male and female patients with Parkinson's Disease (SNCA triplication) and isogenic controls. However, sex was not treated as a primary experimental variable, as the focus of this study was to identify generalizable mechanisms of alpha synuclein–mediated effects on the enteric nervous system (ENS) rather than sex-specific differences. Future studies incorporating sex-stratified analyses may help uncover potential sex-specific contributions to alpha-synuclein-driven pathology in the ENS.

Reporting on race, ethnicity, or other socially relevant groupings

All iPSC lines used in this study, were obtained from caucasian individuals. However, race or ethnicity was not considered a biological variable in the study design, as the focus of this study was to identify generalizable mechanisms of alpha synuclein–mediated effects on the ENS rather than race-specific differences.

Population characteristics

iPSC lines came from one male and two female (aged 37, 49 and 38 respectively). Further information can be obtained from the Coriell NINDS and NIGMS Human Genetic Cell Repositories: GM15010 (3x-1), ND00196 (3x-2), ND00139 (3x-4, referred as 3x-3 throughout this paper) at https://www.coriell.org

The human research participant cohort (gut tissue) consisted of three healthy female controls (ages 25, 48, and 81) and three patients with Ulcerative Colitis (UC; one female aged 47 and two males aged 49 and 60), with UC disease duration ranging from 4 to 15 years.

Recruitment

Human tissue samples were obtained from patients undergoing routine diagnostic or therapeutic endoscopy at the Universitätsklinikum Erlangen. Participants were recruited prospectively based on a clinical need for biopsy; diagnosis of Ulcerative Colitis (UC) was subsequently confirmed by a combination of clinical, endoscopic, radiological, and histological

findings. Written informed consent was obtained from all patients, and data were pseudonymized.

Potential Biases and Impact:A potential self-selection bias exists as only patients seeking medical care at a tertiary referral center were included, which may favor more symptomatic or severe cases of UC. Furthermore, because samples were obtained during routine clinical procedures, the timing of collection relative to disease flares or medication cycles was determined by clinical necessity rather than experimental design. However, given that the study focuses on fundamental cellular mechanisms of alpha-synuclein and MAS enzymes in the enteric nervous system, these biases are unlikely to impact the core molecular findings, though they may reflect a specific subset of disease severity

Ethics oversight

iPSC lines are comercially available, so ethics does not apply. Collection of human gut tissue was approved by the Ethics Committee (#49_16B) of the Universitätsklinikum Erlangen.

Note that full information on the approval of the study protocol must also be provided in the manuscript.

Field-specific reporting

Please select the one below that is the best fit for your research. If you are not sure, read the appropriate sections before making your selection.

☒ Life sciences ☐ Behavioural & social sciences ☐ Ecological, evolutionary & environmental sciences

For a reference copy of the document with all sections, see nature.com/documents/nr-reporting-summary-flat.pdf

Life sciences study design

All studies must disclose on these points even when the disclosure is negative.

Sample size

No statistical methods were applied to calculate sample size in this study. Unless stated otherwise, all quantitative analysis was performed using independently collected material from three isogenic and three SNCA 3x cell lines (our biological replicates, n=3 per genotype). This is in line with previously published studies using the exact same lines (Stojkovska et al, 2022, 10.1016/j.neuron.2021.10.032)

Data exclusions

No data were excluded.

Replication

The number of independent differentiation runs (technical replicates) used to generate the data for each panel is specified in the corresponding figure legend. Each data point in the figures represents the average of these technical replicates for each individual line.

Randomization

The experiments were not randomized; however, to minimize batch effects, SNCA 3x and isogenic lines were processed in parallel across all independent differentiation runs.

Blinding

Blinding was not implemented in this study since the investigator was aware of the disease status of each of the lines used.

Reporting for specific materials, systems and methods

We require information from authors about some types of materials, experimental systems and methods used in many studies. Here, indicate whether each material, system or method listed is relevant to your study. If you are not sure if a list item applies to your research, read the appropriate section before selecting a response.

Materials & experimental systems

n/a	Involved in the study	
☐	☒	Antibodies
☐	☒	Eukaryotic cell lines
☒	☐	Palaeontology and archaeology
☒	☐	Animals and other organisms
☒	☐	Clinical data
☒	☐	Dual use research of concern
☒	☐	Plants

Methods

n/a	Involved in the study	
☒	☐	ChIP-seq
☐	☒	Flow cytometry
☒	☐	MRI-based neuroimaging

Antibodies

Antibodies used

NANOG (APC): RRID: AB_2784439, 1:100, LOT: 5250102275
LIN28A (AF488): RRID: AB_2738303, 1:100, LOT: 4159107
alpha-synuclein 2A7, RRID: AB_1555287,1:100, LOT:092623
HuC/D, RRID: AB_221448, 1:200, LOT: 2566335
GFAP, RRID: AB_10013382, 1:500, LOT: 20035994
Tubulin beta-III, RRID: AB_2313773, 1:250, LOT: B354042
alpha-synuclein 15G7, RRID: AB_11180660, 1:10, LOT: 08182205
aspartate aminotransferase, RRID: AB_3598484, 1:100, LOT: 4000000579
alpha synuclein Syn-1, RRID: AB_398108, 1:1000, LOT: 2182308

β-actin, RRID: AB_2305186, 1:1000, LOT: 1095993-1
CD56, RRID: AB_2565633, 1:100, LOT: B350903
CD24, RRID: AB_2737795, 1:100, LOT: 4099306
CPT1α, RRID: AB_3684993, 1:200, LOT: B424426
alpha-synuclein 2A7 (flow cytometry), RRID: AB_3198368, 1:100, LOT: D170885

Validation

NANOG: Wang Y, et al. (2021) Generation of induced pluripotent stem cell line (ZZUi028-A) from a 52-year-old Chinese Han healthy female individual. Stem cell research, 53, 102381. (PMID:34088010)

LIN28A: QC testing by BD Biosciences. Xiong, K., Zhou, Y., Blichfeld, K. A., Hyttel, P., Bolund, L., Freude, K. K., & Luo, Y. (2017). RNA-Guided Activation of Pluripotency Genes in Human Fibroblasts. Cellular reprogramming, 19(3), 189–198. https://doi.org/10.1089/cell.2017.0006

alpha-synuclein 2A7: Rueda-Gensini, L., Serna, J. A., Rubio, D., Orozco, J. C., Bolaños, N. I., Cruz, J. C., & Muñoz-Camargo, C. (2023). Three-dimensional neuroimmune co-culture system for modeling Parkinson's disease microenvironmentsin vitro. Biofabrication, 15(4), 10.1088/1758-5090/ace21b. https://doi.org/10.1088/1758-5090/ace21b

HuC/D: Gogolou, A., Frith, T. J. R., & Tsakiridis, A. (2021). Generating Enteric Nervous System Progenitors from Human Pluripotent Stem Cells. Current protocols, 1(6), e137. https://doi.org/10.1002/cpz1.137

GFAP: Boreland, A. J., Stillitano, A. C., Lin, H. C., Abbo, Y., Hart, R. P., Jiang, P., Pang, Z. P., & Rabson, A. B. (2024). Sustained type I interferon signaling after human immunodeficiency virus type 1 infection of human iPSC derived microglia and cerebral organoids. iScience, 27(5), 109628. https://doi.org/10.1016/j.isci.2024.109628

Tubulin-beta-III: Each lot of this antibody is quality control tested by formalin-fixed paraffin-embedded immunohistochemical staining. This antibody is well characterized and highly reactive to neuron specific Class III ß-tubulin (ßIII). TUJ1 does not identify ß-tubulin found in glial cells. TUJ1 recognizes an epitope located within the last 15 C-terminal residues. This product has been verified for IHC-F (Immunohistochemistry - frozen tissue sections) on the NanoString GeoMx® Digital Spatial Profiler. The GeoMx® enables researchers to perform spatial analysis of protein and RNA targets in FFPE and fresh frozen human and mouse samples. Fleck, J. S., Jansen, S. M. J., Wollny, D., Zenk, F., Seimiya, M., Jain, A., Okamoto, R., Santel, M., He, Z., Camp, J. G., & Treutlein, B. (2023). Inferring and perturbing cell fate regulomes in human brain organoids. Nature, 621(7978), 365–372. https://doi.org/10.1038/s41586-022-05279-8

alpha-synuclein 15G7: Cuddy, L. K., Wani, W. Y., Morella, M. L., Pitcairn, C., Tsutsumi, K., Fredriksen, K., Justman, C. J., Grammatopoulos, T. N., Belur, N. R., Zunke, F., Subramanian, A., Affaneh, A., Lansbury, P. T., Jr, & Mazzulli, J. R. (2019). Stress-Induced Cellular Clearance Is Mediated by the SNARE Protein ykt6 and Disrupted by α-Synuclein. Neuron, 104(5), 869–884.e11. https://doi.org/10.1016/j.neuron.2019.09.001

aspartate aminotransferase: antibody was validated by the manufacturer using Western-Blotting in several cell lines and immunocytochemistry of HeLa cells.

alpha-synuclein Syn-1: Lee, B., Choi, H. N., Che, Y. H., Ko, M., Seong, H. M., Jo, M. G., Kim, S. H., Song, C., Yoon, S., Choi, J., Kim, J. H., Kim, M., Lee, M. Y., Park, S. W., Kim, H. J., Kim, S. J., Moon, D. S., Lee, S., Park, J. H., Yeo, S. G., ... Yun, S. P. (2024). SARS-CoV-2 infection exacerbates the cellular pathology of Parkinson's disease in human dopaminergic neurons and a mouse model. Cell reports. Medicine, 5(5), 101570. https://doi.org/10.1016/j.xcrm.2024.101570

β-actin: Muñoz, S., Barroso, S., Badra-Fajardo, N., Marqueta-Gracia, J. J., García-Rubio, M. L., Ubieto-Capella, P., Méndez, J., & Aguilera, A. (2024). SIN3A histone deacetylase action counteracts MUS81 to promote stalled fork stability. Cell reports, 43(2), 113778. https://doi.org/10.1016/j.celrep.2024.113778

CD56: Each lot of this antibody is quality control tested by immunofluorescent staining with flow cytometric analysis. Collora JA, Liu R, Pinto-Santini D, et al. Single-cell multiomics reveals persistence of HIV-1 in expanded cytotoxic T cell clones. Immunity. 2022;55(6):1013-1031.e7. doi:10.1016/j.immuni.2022.03.004

CD24: QC tested. Faliti CE, Van TTP, Anam FA, et al. Disease-associated B cells and immune endotypes shape adaptive immune responses to SARS-CoV-2 mRNA vaccination in human SLE. Nat Immunol. 2025;26(1):131-145. doi:10.1038/s41590-024-02010-9

CPT1α: QC tested. Ahl, P.J., Hopkins, R.A., Xiang, W.W. et al. Met-Flow, a strategy for single-cell metabolic analysis highlights dynamic changes in immune subpopulations. Commun Biol 3, 305 (2020). https://doi.org/10.1038/s42003-020-1027-9

Eukaryotic cell lines

Policy information about cell lines and Sex and Gender in Research

Cell line source(s)

B-lymphocytes containing a triplication in the SNCA gene were obtained from the Coriell NINDS and NIGMS Human Genetic Cell Repositories: GM15010 (3x-1), ND00196 (3x-2), ND00139 (3x-4, referred as 3x-3 throughout this paper). Further phenotypic and genotypic details on these lines and subjects are available on https://www.coriell.org under the beforementioned accession numbers. See key resources table for more details. Reprogramming into iPSC was performed by transfection of B-lymphocytes with episomal plasmids containing Oct3/4, L-Myc, Sox2 and Klf4, as previously described

	(Stojkovska et al., 2021). Isogenic controls were generated using a dual nickase CRISPR/Cas9 strategy to disrupt exon 2 of the SNCA gene, fully characterized and validated in a previous publication (Stojkovska et al., 2021).
Authentication	iPSCs were characterized using flow cytometry staining for Nanog and Lin28A. Copy number variation and cell karyotyping analyzes revealed absence of chromosomal abnormalities.
Mycoplasma contamination	All cell lines were tested for mycoplasma contamination every month, and all results were negative during the study.
Commonly misidentified lines (See ICLAC register)	No commonly misidentified cell lines (as per the ICLAC register) were used in this study.

Plants

Seed stocks	n/a
Novel plant genotypes	n/a
Authentication	n/a

Flow Cytometry

Plots

Confirm that:

☒ The axis labels state the marker and fluorochrome used (e.g. CD4-FITC).

☒ The axis scales are clearly visible. Include numbers along axes only for bottom left plot of group (a 'group' is an analysis of identical markers).

☒ All plots are contour plots with outliers or pseudocolor plots.

☒ A numerical value for number of cells or percentage (with statistics) is provided.

Methodology

Sample preparation	For characterization, iPSCs were initially treated with Accutase (Gibco, USA), washed with 1× PBS, centrifuged at 300g at room temperature for 5min and then resuspended in FC buffer (2% FCS, 0.01% sodium azide in PBS). We then counted 500,000 cells per line and proceeded with fixation and permeabilization with 100µl BD Fixation/Permeabilization Solution (BD Bioscience) for 10 minutes, followed by addition of 1ml BD Perm/Wash Buffer (BD Bioscience), incubation for 5 minutes and centrifugation at 300g for 3min. For intracellular staining of iPSCs, we used a combination of Nanog and Lin28A primary antibodies in BD Perm/Wash Buffer for 30 minutes, followed by washing and resuspension in 300µl FC buffer.<br><br>For ENLs, our flow cytometry data analysis was based on the protocol developed by Windster et al. Cells were washed twice with PBS 1x and then incubated with Accutase for 30 min at 37°C and 5% CO_2. Subsequently 2mL of ENC Media was added to inhibit Accutase and cells were centrifuged at 300xg for 3 min, counted and then plated in a 96 well plate. Zombie NIR viability staining dye (Biolegend Cat:423105) was added for 20min (1:2000) followed by two washing steps with FACS buffer (2% FCS, 0.01% sodium azide in PBS). Next, antibodies and/or mitochondrial probes were incubated for 30min at 37°C and 5% CO_2 at the following concentrations: Mitotracker Deep Red, Green and MitoSox at 1:10000 (ThermoFisher); CD56 (Biolegend, RRID: AB_2565633, 1:100), CD24 (BD Biosciences, RRID: AB_2737795, 1:100), CPT1α (BD Biosciences, RRID: AB_3684993, 1:200). Cells were acquired in the MACSQuant Analyzer 16 Flow Cytometer (Miltenyi). Results were anaylzed in FlowJo 10.1 where the dimensional reduction analysis was also employed. For this, we used unsupervised tSNE algorithm where neuronal (CD56+CD24high) and glial (CD56+CD24low) cells were concatenated and submitted to tSNE analysis. Plots were then generated and populations selected based on the markers of interest.
Instrument	Flow cytometry was performed using a CytoFLEX flow cytometer (Beckman Coulter) and MACSQuant Analyzer 16 Flow Cytometer (Miltenyi).
Software	Data were analyzed using CytExpert software (Beckman Coulter) and FlowJo 10.1 (BD Biosciences)
Cell population abundance	No cells were sorted, only flow cytometry analysis was performed. The abundances of the individual populations are shown in the respective graphs.
Gating strategy	The gating strategy began with debris exclusion using FSC-Width and FSC-H parameters. Single cells were identified by excluding doublets through gating on FSC-A versus FSC-H plots. Viability was determined using the ombie NIR viability staining dye , allowing for exclusion of dead cells. Finally, populations of interest were identified based on the expression of fluorescently labeled surface and intracellular markers. For ENLs, we added an extra step by separating between enteric neurons (CD56+CD24high) and enteric glia (CD56+CD24low) for downstream analyzes. For identifying positive populations,

fluorescence intensity thresholds were determined using fluorescence-minus-one (FMO) controls, isotype controls and/or unstained samples to set boundaries between negative and positive populations. Populations expressing specific markers were selected based on clear shifts in fluorescence intensity compared to these controls. Compensation was applied to correct for spectral overlap between fluorophores, ensuring accurate identification of populations. Each marker's positive population was defined as the cell subset showing fluorescence above the threshold set by the respective control.

☒ Tick this box to confirm that a figure exemplifying the gating strategy is provided in the Supplementary Information.
